# MiR-613 inhibits proliferation and invasion and induces apoptosis of rheumatoid arthritis synovial fibroblasts by direct down-regulation of DKK1

**DOI:** 10.1186/s11658-018-0130-0

**Published:** 2019-04-19

**Authors:** Liang Liu, Yanhua Zuo, Yan Xu, Zongfang Zhang, Ying Li, Jie Pang

**Affiliations:** 1 Department of Rheumatology and Immunology, Cangzhou Central Hospital, Cangzhou, 061000 People’s Republic of China; 2 The Second Nephrology Department, Cangzhou Central Hospital, Cangzhou, 061000 People’s Republic of China

**Keywords:** Rheumatoid arthritis, MicroRNA-613, Rheumatoid arthritis synovial fibroblasts, DKK1, Proliferation, Apoptosis

## Abstract

**Background:**

This study aimed to investigate the effects of miR-613 on the proliferation, invasion and apoptosis of rheumatoid arthritis synovial fibroblasts (RASFs).

**Methods:**

Synovial tissue samples were collected from 20 rheumatoid arthritis (RA) patients and 10 patients with joint trauma undergoing joint replacement surgery. The RASFs were isolated and cultured. MiR-613 and DKK1 expression in both synovial tissues and cells was detected using quantitative real-time PCR (qRT-PCR). Dual luciferase reporter gene assay was employed to evaluate the effect of miR-613 on the luciferase activity of DKK1. Then RASFs were transfected with miR-613 mimics, si-DKK1 and pcDNA-DKK1. Changes in cellular proliferation, invasion and apoptosis were detected through BrdU assay, Transwell invasion assay and flow cytometry analysis, respectively.

**Results:**

MiR-613 was significantly down-regulated in RA tissues and RASFs compared to normal tissues and cells, whereas DKK1 was up-regulated in RA tissues and RASFs. Dual luciferase reporter gene assay showed that miR-613 could specifically bind to the 3′UTR of DKK1 and significantly inhibit the luciferase activity. Moreover, miR-613 significantly reduced the expression of DKK1. Overexpression of miR-613 or knockdown of DKK1 suppressed proliferation and invasion of RASFs, and induced RASF apoptosis. The reverse results were observed when DKK1 was up-regulated in miR-613-overexpressing RASFs.

**Conclusions:**

MiR-613 can inhibit proliferation and invasion and induce apoptosis of RASFs by directly targeting DKK1 expression.

## Background

Rheumatoid arthritis (RA), as one of the most common inflammatory diseases, affects more than 1% of the general population worldwide. It is characterized by inflammation, synovitis and damage of articular cartilage as well as bone, and if not treated effectively 40–70% of patients will eventually progress to disability [1, 2]. Apart from joint involvement, patients with longer disease duration can have various extraarticular manifestations such as interstitial lung disease and cardiovascular disease [3, 4]. These greatly increase the disease and social burden in RA patients, which necessitates early diagnosis and sufficient treatment in RA management [5].

DKK1 is a Wingless (Wnt) signaling pathway inhibitor, and it has been considered as a master regulator of joint remodeling [6]. It was reported that joint erosions and inflammation were positively correlated with serum levels of DKK1 in RA [7]. Indeed, higher DKK1 levels were observed in serum of patients with a genetic variant of DKK1 and resulted in having more progressive joint destruction [8], suggesting a fundamental role for DKK1 in the pathogenesis of RA. The loss of bone in murine models of arthritis can be restored by treatment with antibodies against DKK1 [6], indicating that DKK1 has promise as a novel therapeutic target. In this study, to determine the role of DKK1 in the pathogenesis of RA, we analyzed, for the first time to our knowledge, the expression of DKK1 in RA synovial fibroblasts (RASFs) compared with patients with joint trauma undergoing joint replacement surgery (healthy controls). Knockdown of DKK1 significantly suppressed proliferation and invasion of RASFs, and induced RASF apoptosis.

Recently, it has been increasingly reported that microRNAs (miRNAs) play an important role in the pathogenesis of RA. It is widely known that miRNAs negatively regulate gene expression by binding to the 3′-untranslated region (3′-UTR) of their target mRNAs, resulting in degradation of mRNAs or down-regulation of the corresponding proteins. For example, Xu et al. reported that miR-650 can inhibit proliferation, migration and invasion of RASFs by targeting AKT2 [9]. Moreover, a recent study demonstrated that miR-126 affects RASF proliferation and apoptosis through the PI3K-AKT signaling pathway by targeting PIK3R2 [10]. In this study, we particularly focus on miR-613 and its role in the pathogenesis of RA. We thus speculated that miRNA-613 might modulate RA development by binding to its target mRNAs, which thereby leads to the loss of the functions mediated by the corresponding proteins. For the first time, we found that DKK1 down-regulation could significantly inhibit the proliferation and invasion and induce apoptosis of RASFs. Moreover, miR-613 overexpression also suppressed proliferation and invasion and induced apoptosis of RASFs by directly targeting DKK1.

## Materials and methods

### Tissue specimen collection

Synovial tissue samples from 20 RA patients (12 male and 8 female, 33–67 years old, mean 51) were obtained during joint surgery at Cangzhou Central Hospital from 2016 to 2017. All RA patients fulfilled the American College of Rheumatology criteria for classification of disease [11]. Healthy control specimens (6 male and 4 female, 31–65 years old, mean 48) were obtained from patients with joint trauma undergoing joint replacement surgery at Cangzhou Central Hospital from 2016 to 2017. Healthy control specimens were free of other diseases such as autoimmune disease, infectious disease and cancer. This study was approved by the Ethical Committee of Cangzhou Central Hospital (2016021621) and complied with the guidelines and principles of the Declaration of Helsinki. All participants signed written informed consent.

### Cell line and cell culture

Human RASFs were isolated and cultured as previously described [12]. Synovial tissues were taken out intraoperatively and cut into pieces immediately under sterile conditions. The cut synovial tissues were subjected to digestion by 2.5 g/L trypsin at 37 °C for 2 h. After that, the digested synovial tissues were subjected to centrifugation to obtain RASFs. RASFs at passages 3–8 were subjected to experiments. RASFs were maintained in Dulbecco’s modified Eagle’s medium (DMEM, Invitrogen, USA) supplemented with 10% fetal bovine serum (FBS, Gibco, USA) and penicillin and streptomycin (P/S, Gibco) at 37 °C within 5% CO_2_. Cells were grown in 6-well plates with 75% confluence at 24 h before transfection.

### Transient transfection

The miR-613 mimic, miR-negative control (miR-NC), siRNA for DKK1 (si-DKK1, 5′-TGATAGCCCTGTACAATGCTGCT-3′) and siRNA-negative control (si-NC) were synthesized and purified by Gene-Pharma (Shanghai, China). The DKK1-overexpression plasmid was generated by inserting DKK1 cDNA into a pcDNA3.1 vector. The sequence of this plasmid was confirmed by Gene-Pharma. The miR-613 mimic, miR-NC, si-DKK1 and DKK1-overexpression plasmid were transfected into the RASFs according to the instructions of the purchased Lipofectamine RNAIMAX transfection kit (Invitrogen, USA). The detailed procedures were as follows: (1) 1 day before transfection, the RASFs were plated into 6-well plates at the concentration of 1 × 10^6^ cells per well; (2) on the day of transfection, the Lipofectamine RNAIMAX transfection reagent was mixed evenly with opti-MEM culture medium and the synthesized miR-613 mimic, miR-NC or si-DKK1, and the mixtures were incubated for 5–10 min at room temperature before they were added into the cell culture medium; (3) 48 h after the transfection, cells were digested with trypsin, rinsed once with PBS, and preserved for further experiments.

### ELISA-BrdU assay

To investigate the effects of the miR-613 mimic and si-DKK1 on cell proliferation of RASFs, the ELISA-BrdU assay was selected to detect cell proliferation using the Cell Proliferation ELISA-BrdU Kit (Roche Applied Science, Mannheim, Germany) following the manufacturer’s instructions. Briefly, 5 × 10^3^ cells were seeded in a 96-well plate (Corning, USA) and allowed to grow overnight in complete medium. The medium was then removed and the cells were transfected with the miR-613 mimic or miR-NC for 48 h at 37 °C. After 48 h incubation, cells were additionally treated with BrdU labeling solution for a further 16 h. After that, culture medium was removed, cells were fixed and DNA was denatured. Cells were incubated with anti-BrdU-POD solution for 90 min, and then antibody conjugates were removed by washing three times. After incubation with a TMB substrate for 15 min, absorbance at 405 and 490 nm was measured to determine immune complexes.

### RNA extraction and real-time quantitative PCR

Total RNA was extracted from cell lines and clinical samples by using TRIzol Reagent (Invitrogen, Carlsbad, CA, USA) according to the operating instructions. RNA was quantified by using UV absorbencies at 260 and 280 nm (A260/280). Subsequently the RNA was reverse-transcribed into cDNA using a reverse transcription system (Thermo Scientific, CA, USA). The level of miR-613 was detected by the ABI PRISM 7500 Sequence Detection System (ABI) using the TaqMan MicroRNA assay kits (Applied Biosystems, California, USA). U6 small nuclear RNA (snRNA) was used as the normal control. The mRNA expression levels of DKK1, MMP-2, and MMP-9 were also analyzed by SYBR Green and normalized to GAPDH. The judgment of primer sequences’ specificity was based on the dissociation curve, and 2^-ΔΔCt^ (cycle threshold) was used to calculate the relative gene expression levels. Primer sequences are shown in Table 1.

**Table 1:** Sequence of primers for qRT-PCR

Gene	Primer sequence
DKK1	F: 5’-CCTTGAACTCGGTTCTCAATTCC-3′
	R: 5’-CAATGGTCTGGTACTTATTCCCG-3′
MMP-2	F: 5’-CTGCGGTTTTCTCGAATCCA-3′
	R: 5’-GGGTATCCATCGCCATGCT-3’
MMP-9	F: 5’-CCCTGGAGACCTGAGAACCA-3’
	R: 5′- CCACCCGAGTGTAACCATAGC-3’
U6	F: 5’-CTCGCTTCGGCAGCACA-3’
	F: 5’-AACGCTTCACGAATTTGCGT-3’
GAPDH	F: 5’-GAGTCAACGGATTTGGTCGTATTG-3’
	R: 5’-CCTGGAAGATGGTGATGGGATT-3’

### Apoptosis assay

Cell apoptosis was detected by the Annexin V-FITC/PI Kit (Beijing Biosea Biotechnology, Beijing, China), according to the manuals. Briefly, the cells (100,000 cells/well) were seeded in a 6 well-plate. Treated cells were washed twice with cold phosphate buffer saline (PBS) and resuspended in buffer. The adherent and floating cells were combined and treated according to the manufacturer’s instructions and measured with a flow cytometer (Beckman Coulter, USA) to differentiate apoptotic cells (Annexin V positive and PI-negative) from necrotic cells (Annexin V and PI-positive).

### Transwell invasion assay

Transwell Matrigel invasion assay using Transwell chambers (8-mm pore size; Minipore) precoated with Matrigel (BD Biosciences, Franklin Lakes, NJ) that contained extracellular matrix proteins was used to determine cell invasion. In brief, 1 × 10^5^ cells in 100 μl of DMEM containing 1% FBS were seeded in the upper chamber, and 600 μl of DMEM containing 10% FBS was added to the lower chamber. After 6 h incubation at 37 °C in a 5% CO_2_ atmosphere, cells that remained in the upper chamber were removed by cotton swabs and penetrating cells were fixed in methanol, and then stained with 0.1% crystal violet. Cells were imaged from at least five grids per field. Then the membranes were rinsed with 30% glacial acetic acid. Finally, the wash solution was examined at 540 nm to count the number of glioma cells. All assays were independently repeated three times.

### Western blot analysis

RASFs were washed twice in cold PBS, and then lysed in RIPA lysis buffer (Beyotime Institute of Biotechnology Jiangsu, China). The protein concentration of cell lysates was quantified by a BCA Kit (Beyotime Institute of Biotechnology Jiangsu, China), and 50 μg of each protein were separated by SDS-PAGE on 10% gels, and then transferred to a polyvinylidene fluoride (PVDF) membrane (Millipore, USA). The membranes were blocked in 5% non-fat milk diluted with Tri Buffered Saline Tween-20 (TBST) at room temperature for 1 h and incubated overnight at 4 °C with primary antibody: anti-p21, anti-cyclin D1, anti-CDK4, anti-Bax (1:500; Cell Signaling Technology Inc., MA, USA); anti-DKK1, anti-PCNA, anti-MMP-2, anti-MMP-9 (1:1000; Abcam, USA). The membranes were then incubated with goat anti-rabbit or anti-mouse IgG conjugated to horseradish peroxidase secondary antibody (1:1000; Cell Signaling Technology Inc., MA, USA) for 2 h. The proteins were visualized using ECL-plus reagents (Amersham Biosciences Corp., USA). The density of the bands was measured using the Image J software (USA), and values were normalized to the densitometric values of α-tubulin (1:5000; Sigma, USA) in each sample.

### Measurement of MMP-2 and MMP-9 levels

Enzyme-linked immunosorbent assay (ELISA) kits (USCN, USCN life science, Wuhan, China) were used to determine the levels of MMP-2 and -9 in the culture supernatants based on the manufacturer’s instructions.

### Luciferase reporter assay

RASFs were seeded in 24-well plates and incubated for 24 h before transfection. The pGL3-DKK1–3′UTR wild-type or mutant plasmid was cotransfected with the miR-613 mimic or miR-NC, and pRL-SV40 Renilla plasmid (Promega, USA) into RASFs. After transfection for 48 h, both firefly and Renilla luciferase activities were detected by a dual-luciferase reporter system (Promega, USA) following the manufacturer’s protocols. All experiments were performed in triplicate.

### Statistical analysis

All statistical analyses were performed using GraphPad Prism 5.0 (GraphPad Software, Inc., USA). Data from each group were expressed as mean ± standard error of the mean (S.E.M.) and statistically analyzed by Student’s t test. Differences were considered statistically significant at a *p* value of < 0.05.

## Results

### The level of DKK1 miR-613 is down-regulated in synovial tissues and RASFs

**Fig. 1:**
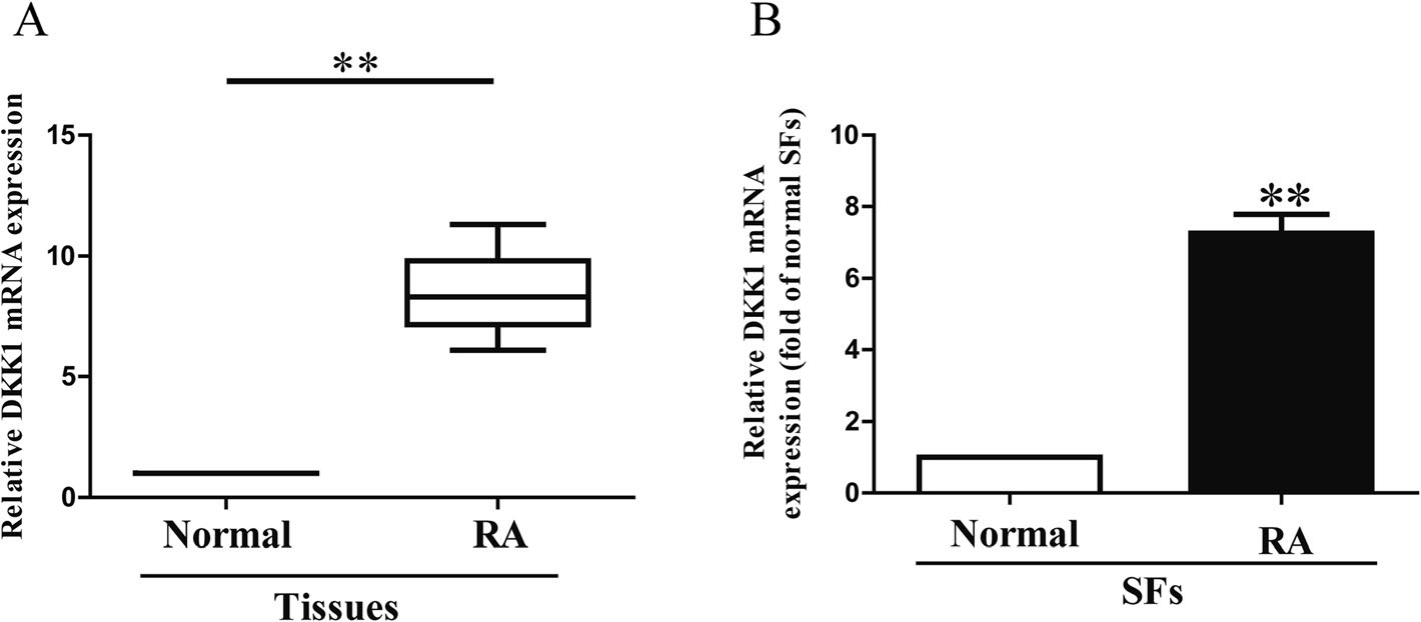
Level of DKK1 in RA tissues and SFs. (**a**) Relative DKK1 expression levels in RA tissues and their corresponding adjacent normal tissues. (**b**) Relative DKK1 level analyzed by RT-PCR in RASFs and their corresponding adjacent normal SFs normalized with U6 snRNA. All data are presented as mean ± SEM, *n* = 6. **P < 0.01 vs. normal tissues or SFs

It has been reported that the level of DKK1 was significantly up-regulated in synovial fibroblasts from *patients* [13]. However, the role of DKK1 in synovial fibroblasts remains unknown. In this study, we also found that the expression of DKK1 in synovial tissues from RA patients was significantly increased in comparison to the adjacent normal tissues (Fig. 1a). Next, we further confirmed the enhanced expression of DKK1 in RASFs (Fig. 1b).

### Knockdown of DKK1 significantly inhibited cell proliferation and invasion and promoted apoptosis in RASFs

**Fig. 2:**
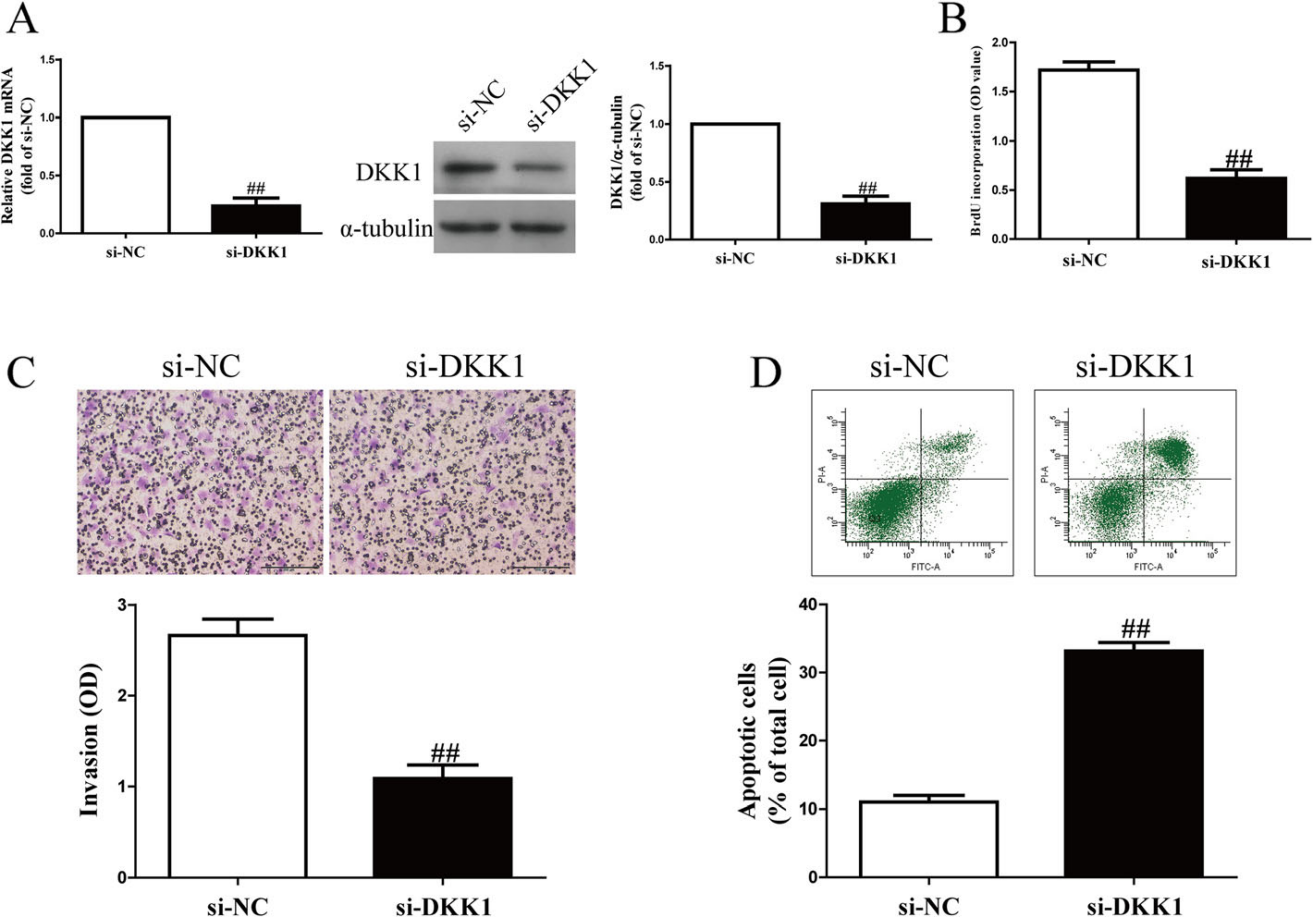
Effects of DKK1 silencing on cell proliferation, invasion and apoptosis in RASFs. RASFs were transfected with si-DKK1 or si-NC for 48 h. (**a**) Protein and mRNA expression of DKK1 was determined by qRT-PCR and Western blot, respectively. (**b**) Cell proliferation was assessed by BrdU-ELISA assay. (**c**) Invasion was assessed by Transwell assay after 6 h. (**d**) Cell apoptosis was measured by flow cytometric analysis of cells labeled with Annexin-V/PI double staining. All data are presented as mean ± SEM, *n* = 6. ^##^*P* < 0.01 vs. si-NC

To study the effects of DKK1 on RASFs, cell proliferation, invasion and apoptosis were estimated in RASFs after transfection with si-NC or si-DKK1 for 48 h. Western blot and qRT-PCR analysis showed that the DKK1 expression was significantly decreased in RASFs after transfection with si-DKK1 for 48 h compared to the si-NC group (Fig. 2a). The BrdU-ELISA assay indicated that knockdown of DKK1 could significantly suppress the proliferation of RASFs (Fig. 2b). Furthermore, the Transwell assays suggested that decreased DKK1 expression inhibited invasive ability of RASFs (Fig. 2c). Finally, knockdown of DKK1 promoted apoptosis of RASFs (Fig. 2d).

### miR-613 directly targeted DKK1 3′UTR

**Fig. 3:**
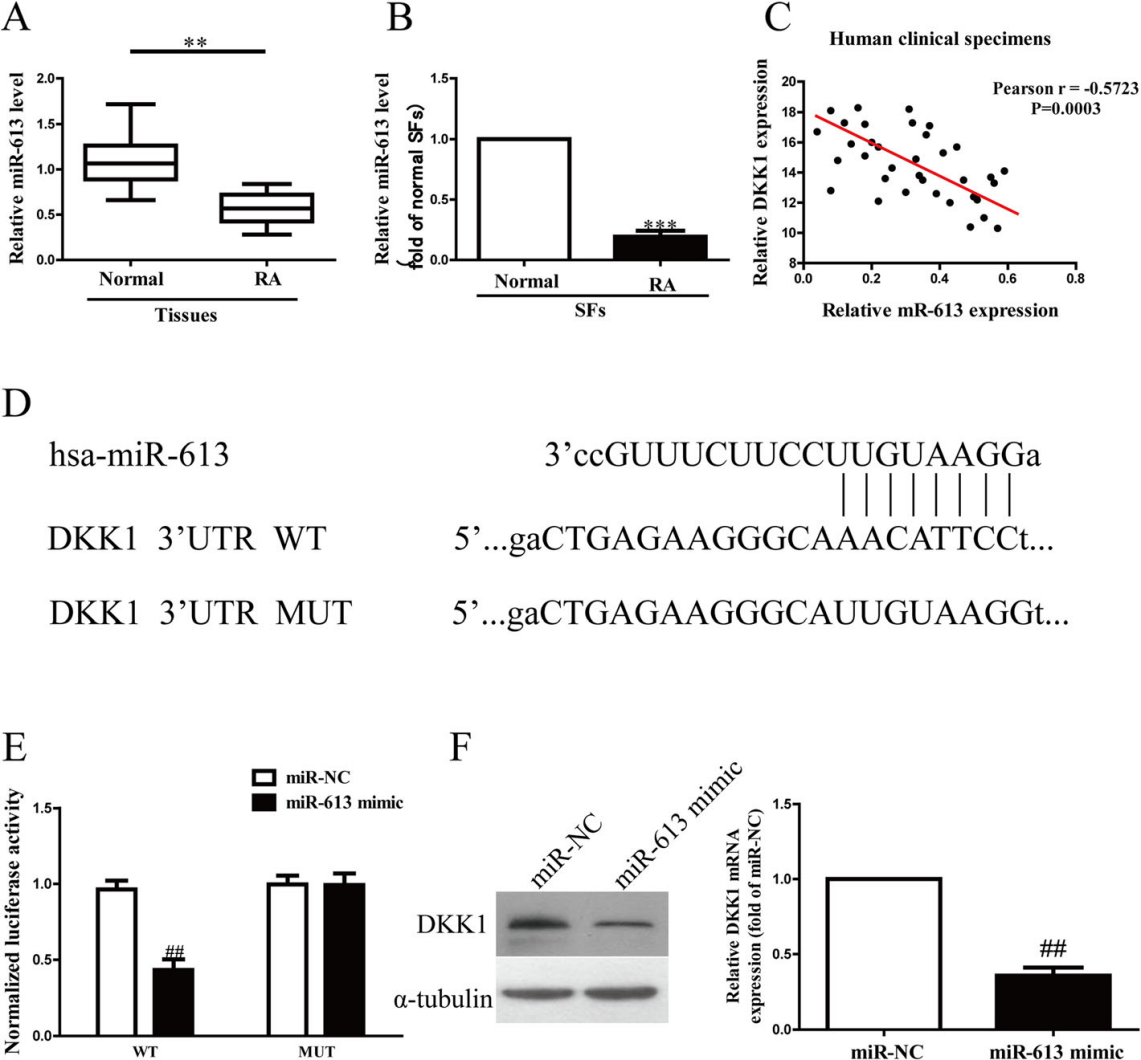
DKK1 was a direct target of miR-613. RASFs were transfected with miR-613 mimic or miR-NC for 48 h. (**a**) Relative miR-613 level in RA tissues and their corresponding adjacent normal tissues. (**b**) Relative miR-613 level analyzed by RT-PCR in RASFs and their corresponding adjacent normal SFs were normalized with U6 snRNA. (**c**) Pearson’s correlation analysis of relative expression levels of miR-613 and relative DKK1 mRNA levels in RA tissues. (**d**) Schematic representation of DKK1 3′UTRs showing putative miRNA target site. (**e**) Analysis of relative luciferase activities of DKK1-WT and DKK1-MUT. (**f**) Protein and mRNA expression of DKK1 was determined by Western blot and qRT-PCR, respectively. All data are presented as mean ± SEM, *n* = 6. **P < 0.01, ***P < 0.001 vs. normal tissues or SFs; ^##^*P* < 0.01 vs. miR-NC

For further study, the online database microRNA.org predicted that miR-613 might directly target DKK1. Our data confirmed that the miR-613 level in synovial tissues from RA patients was markedly lower than that in the adjacent normal tissues (Fig. 3a). To support this result, we also demonstrated that the miR-613 level was significantly decreased in RASFs, as shown in Fig. 3b. To study whether the DKK1 expression was closely associated with miR-613 in synovial tissues from RA patients or not, the Pearson’s correlation analysis revealed a significant inverse correlation between DKK1 and miR-613 in synovial tissues from RA patients (Fig. 3c).

According to the online database microRNA.org, we identified a miR-613 binding site in the 3′UTR of DKK1 (Fig. 3d). To validate whether DKK1 is a direct target of miR-613, luciferase plasmids containing the potential DKK1 miR-613 binding sites (WT) or a mutated DKK1 3′UTR were constructed (Fig. 3d). Overexpression of miR-613 inhibited WT DKK1 reporter activity but not the activity of the mutated reporter construct in RASFs, demonstrating that miR-613 could specifically target the DKK1 3′UTR by binding to the seed sequence (Fig. 3e). Next, we confirmed that introduction of miR-613 could significantly decrease the expression of DKK1 (Fig. 3f). These data indicated that miR-613 directly regulated DKK1 expression in RASFs through 3′-UTR sequence binding.

### Effects of miR-613 overexpression on cell proliferation, cell cycle and apoptosis in RASFs

**Fig. 4:**
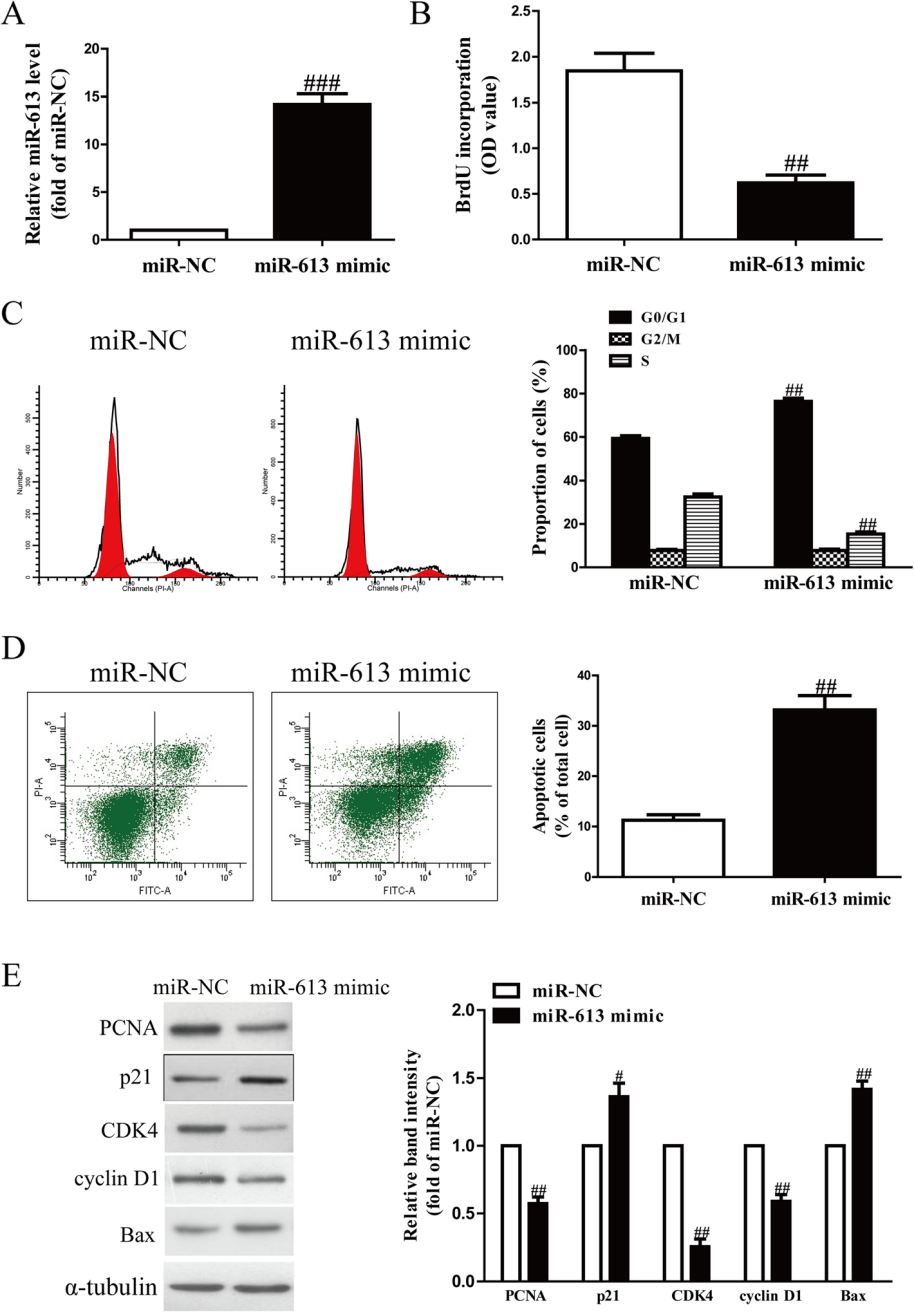
Introduction of miR-613 inhibited cell proliferation, arrested cell cycle and induced cell apoptosis in RASFs. RASFs were transfected with miR-613 mimic or miR-NC for 48 h. (**a**) mRNA level of miR-613 in RASFs was determined by qRT-PCR. (**b**) Cell proliferation was assessed by BrdU-ELISA assay. (**c**) Cell cycle was detected by flow cytometry. (**d**) Cell apoptosis was measured by flow cytometric analysis of cells labeled with Annexin-V/PI double staining. (**e**) mRNA expression of PCNA, p21, CDK4, cyclin D1 and Bax was determined by Western blot. All data are presented as mean ± SEM, n = 6. ^#^*P* < 0.05, ^##^P < 0.01, ^###^P < 0.001 vs. miR-NC

Next, we evaluated whether miR-613 could affect cell proliferation, cell cycle and apoptosis of RASFs. After transfection with the miR-613 mimic or miR-NC, we found that the level of miR-613 in RASFs was significantly increased in the miR-613 mimic group compared to the miR-NC group (Fig. 4a). The results from the BrdU-ELISA assay to explore the role of miR-613 in RASF proliferation demonstrated that up-regulation of miR-613 had an anti-proliferative effect in RASFs (Fig. 4b).

Since the miR-613 mimic significantly suppressed RASF proliferation, we speculated that introduction of miR-613 could arrest the cell cycle of RASFs. Our flow cytometry results demonstrated that overexpression of miR-613 dramatically increased the percentage of cells in the G1-phase in both RASFs compared with cells transfected with miR-NC (Fig. 4c). Therefore, overexpression of miR-613 might inhibit proliferation of RASFs by hindering the transition of the cell cycle from G1 phase to S phase.

In order to further study whether the miR-613 mimic exerted its anti-proliferative effect through induction of cell apoptosis, the total apoptosis rates of RASF cells were also detected by flow cytometry analysis. We confirmed that the apoptotic rate of RASFs was higher in the miR-613 mimic group than in the miR-NC group (Fig. 4d).

To confirm these effects at the molecular level, related proteins including PCNA (a proliferation marker), p21 protein (a cyclin-dependent kinase inhibitor), CDK4 (a cyclin-dependent kinase), cyclin D1 (a cell cycle protein), and Bax (a pro-apoptotic protein) were determined by Western blot analysis. We found that the expression of PCNA, CDK4 and cyclin D1 displayed obvious down-regulation in the miR-613 mimic group compared to the miR-NC group (Fig. 4e). However, expression of p21 and Bax proteins was significantly up-regulated by overexpression of miR-613 (Fig. 4e). These findings suggested that introduction of miR-613 might be associated with down-regulation of PCNA, CDK4 and cyclin D1, and up-regulation of Bax and p21 in RASFs.

### Introduction of miR-613 inhibited invasion and expression of related molecules in RASFs

**Fig. 5:**
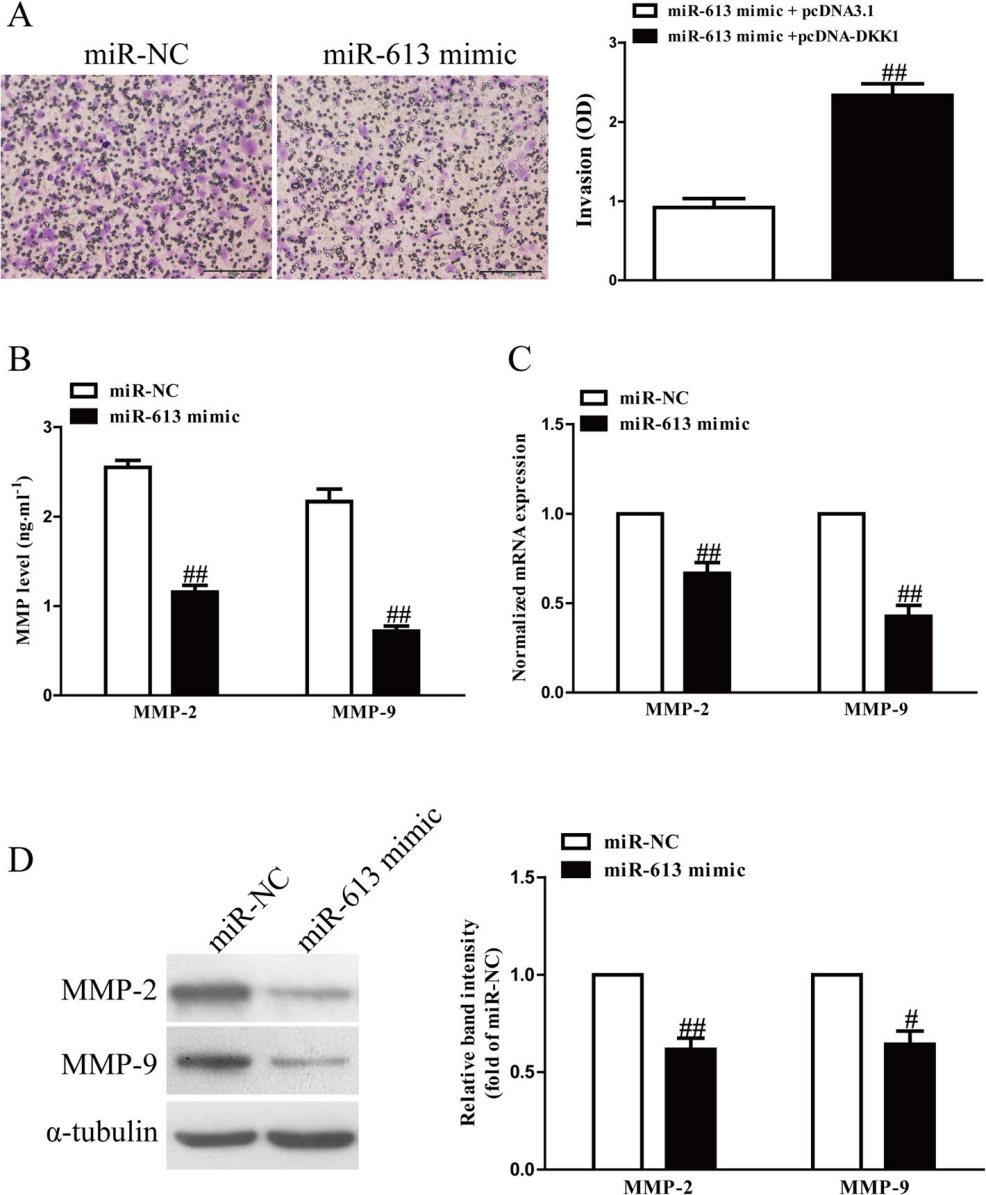
Up-regulation of miR-613 suppressed invasion of RASFs. RASFs cells were transfected with miR-613 or miR-NC for 48 h, and then seeded in the top chamber. (**a**) After 6 h, the RASFs that invaded through the membrane were stained and quantified. (**b**) Levels of MMP-2 and -9 were detected in the culture supernatants of cultured RASFs by ELISA assay. (**c**) mRNA levels of MMP-2 and -9 were examined by qRT-PCR. (**d**) Protein expression of MMP-2 and -9 was determined by Western blotting. All data are presented as mean ± SEM, *n* = 6. ^#^*P* < 0.05, ^##^*P* < 0.01 vs. miR-NC

To determine the function of miR-613 in invasion of RASFs, we evaluated the invasive capacities of RASFs transfected with the miR-613 mimic by Transwell invasion assays. The data from Transwell assays showed that the invasion capability of RASFs was significantly inhibited in the miR-613 mimic group compared to miR-NC group (Fig. 5a). These results showed that miR-613 might play a critical role in suppression of invasion in RASFs.

MMPs may be responsible for the impaired invasion of anti-miR-613-transfected cells. To confirm this hypothesis, we used an ELISA kit to detect the levels of MMP-2 and MMP-9 in the culture supernatants. Our data indicated that secretions of MMP-2 and -9 in the culture supernatants were evidently decreased in miR-613-overexpressed RASFs (Fig. 5b). Additionally, we further detected the expression of MMP-2 and -9 at the mRNA level by RT-PCR assay. After transfection with the miR-613 mimic, MMP-2 and -9 expression at the mRNA and protein levels was distinctly reduced (Fig. 5c, d). Our results suggested that down-regulation of MMP-2 and -9 might be one of the possible mechanisms contributing to the inhibitory effect of the miR-613 mimic on the invasive capacities of RASFs. Consequently, miR-613 overexpression had similar effects as DKK1 silencing on RASFs.

### Up-regulation of DKK1 partially blocked the effect of miR-613 overexpression on RASFs

**Fig. 6:**
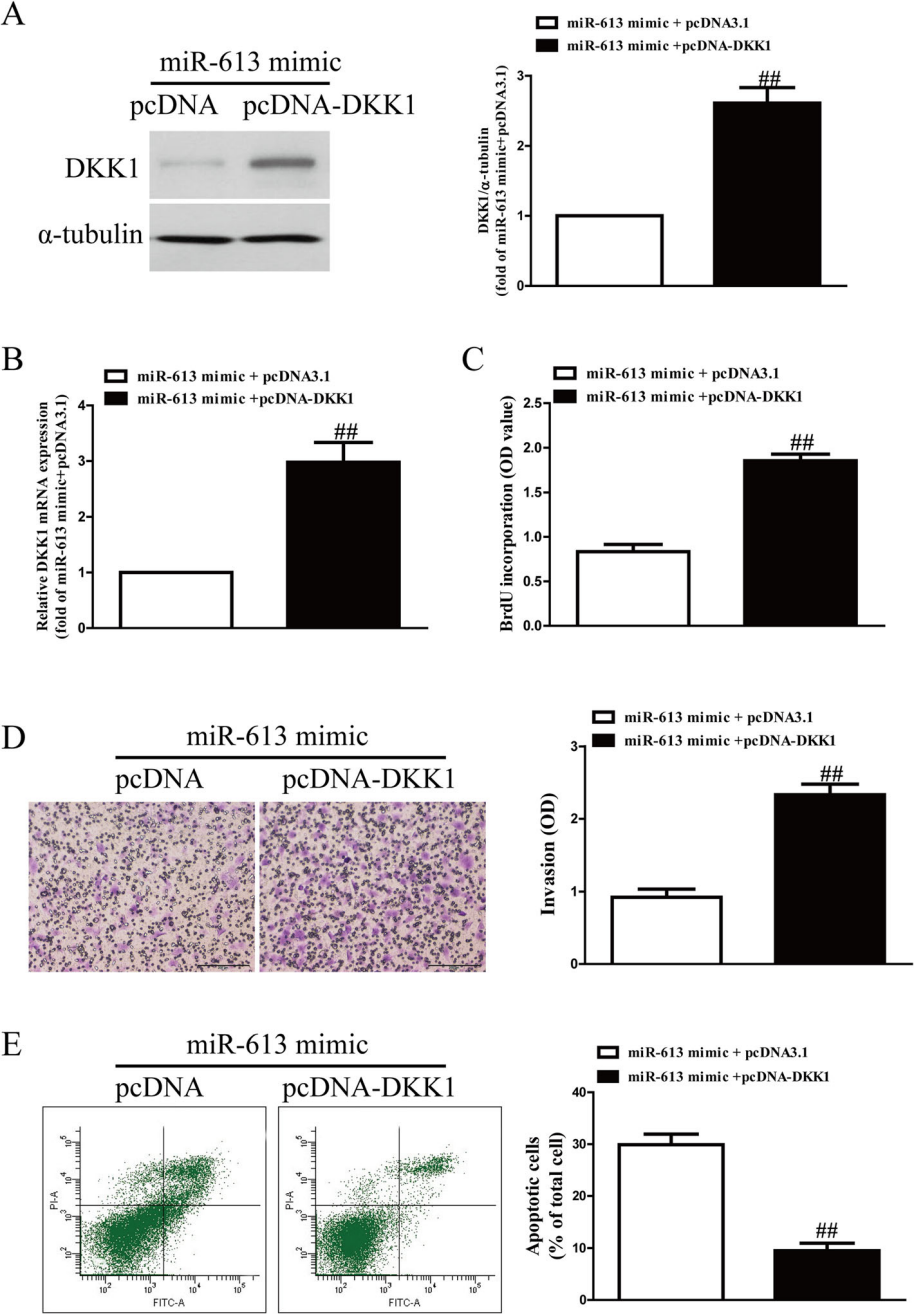
Overexpression of DKK1 partially blocked the effects of miR-613 mimic on cell proliferation, invasion and apoptosis in RASFs. RASFs were transfected with miR-613 mimic either with or without pcDNA3.1-DKK1 vector. (**a**) Protein expression of DKK1 was determined by Western blot. α-tubulin was detected as a loading control. (**b**) mRNA level of DKK1 was determined by qRT-PCR. (**c**) Cell proliferation was assessed by BrdU-ELISA assay. (**d**) Invasion of RASF cells was assessed by Transwell assay after 6 h. (**e**) Cell apoptosis was measured by flow cytometric analysis of cells labeled with Annexin-V/PI double staining. All data are presented as mean ± SEM, *n* = 6. ^##^*P* < 0.01 vs. miR-613 mimic + pcDNA3.1

To confirm whether miR-613 affected RASFs by directly down-regulating DKK1, we cotransfected RASFs with the miR-613 mimic and pcDNA-DKK1. Overexpression of DKK1 significantly enhanced the DKK1 expression inhibited by the miR-613 mimic (Fig. 6a). Results from the BrdU-ELISA assay showed that introduction of DKK1 ignificantly increased cell proliferation of RASFs transfected with the miR-613 mimic (Fig. 6b). Furthermore, the Transwell assay showed that increased DKK1 expression could reverse the inhibitory effect of the miR-613 mimic on invasion of RASFs (Fig. 6c). Moreover, overexpression of DKK1 inhibited the apoptosis of RASFs induced by miR-613 overexpression (Fig. 6d). Therefore, the effects of the miR-613 mimic were partially reversed by DKK1 overexpression. Our data clearly showed that miR-613 inhibited cell proliferation and invasion and promoted apoptosis in RASFs by directly down-regulating DKK1 expression.

## Discussion

Previous studies have reported that DKK1 directly impairs osteoblast differentiation and indirectly enhances bone destruction by promoting RANKL-induced osteoclastogenesis [6, 14]. In established RA, expression of DKK1 within the synovium localizes to synovial fibroblasts ex vivo [6] and is tightly regulated by glucocorticoid metabolism in vitro [15], supporting a role for Wnt signaling inhibition in RA bone destruction. Juarez et al. demonstrated that differential expression of DKK1 was detected in resolving and early RA [13], which suggested that increased DKK1 expression could be a key event in progression to RA and occurs early in the disease process. They also confirmed that Wnt signaling inhibition by DKK1 may therefore be an as yet undefined pathway through which synovial fibroblasts influence bone destruction in early RA [13]. In this study, the expression of DKK1 was significantly increased in RA tissues and cells. Moreover, we found that silencing DKK1 could inhibit RASF proliferation and invasion and promote RASF apoptosis. Altogether, these results suggested that DKK1 had a critical role in RA pathogenesis.

Previous reports have demonstrated that miRNAs play a multifunctional role in several biological processes such as cell proliferation, differentiation, apoptosis, migration, and invasion during inflammation and abnormal innate immune responses [16–18], which makes them potential targets in treatment of numerous autoimmune diseases. Increasing evidence has indicated that miRNAs are closely associated with the pathological progression of RA [19]. Previous reports have demonstrated that the levels of miR-522 [20], miR-140-5p [21], miR-20a [22], miR-338-5p [23], miR-155 [24], miR-125b [25], and miR-29a [26] were altered in synovial fibroblasts and FLSs from RA patients, indicating their roles as RA agonists or suppressors in RA pathogenesis. Therefore, the alteration of miRNA expression could be a marker and shed light on a novel therapeutic strategy in the treatment of RA [19]. In this study, miR-613 was found decreased in RASFs, suggesting that miR-613 might be involved in the regulation of RA pathogenesis.

Generally, miRNAs mediate multiple biological processes through different target sites and regulate the expression of their downstream mRNA targets [27–29]. Previous studies have identified many target mRNAs of miRNA-613, including phosphatase non-receptor type 9 (PTPN9), the sex-determining region Y-box 9 (SOX9), and sphingosine kinase 1 (SphK1) [30–32]. For example, MiR-613 suppressed laryngeal squamous cell carcinoma progression through regulating PDK1 [33]. MicroRNA-613 impedes the proliferation and invasion of glioma cells by targeting cyclin-dependent kinase 14 [34]. MiR-613 functions as a tumor suppressor in hepatocellular carcinoma by targeting YWHAZ [35]. miR-613 inhibits cell migration and invasion by downregulating Daam1 in triple-negative breast cancer [36]. CXCR4-mediated osteosarcoma growth and pulmonary metastasis is suppressed by microRNA-613 [37]. Using the microRNA.org database, it was predicted that DKK1 could be a potential target of miR-613. We conducted a luciferase reporter assay to test whether miR-613 binds to the 3’UTR of the DKK1 gene. Our results showed that DKK1 is a target of miR-613. We also showed that DKK1 mRNA and protein levels in RASFs transfected with the miR-613 mimic were lowered compared to those in control cells. According to our results, overexpression of miR-613 significantly inhibited RASF proliferation and invasion and promoted apoptosis of RASFs. Moreover, overexpression of DKK1 significantly reversed the effects of the miR-613 mimic on RASFs. It was reported that excess RASFs promoted joint destruction [38]. Recent evidence suggests that RASFs secrete excess matrix destructive enzymes as well as proinflammatory cytokines/chemokines, which then promotes joint destruction [39, 40]. Taken together, our findings indicated that miR-613 played a protective role in RA by promoting the death of excess RASFs. The elimination of excess RASFs could lead to the alleviation of RA progression.

## Conclusions

In conclusion, we assessed and reported, for the first time, the effect of miR-613 and its target gene DKK1 on RASFs and elucidated the possible pathological progression of RA. Although we consider miR-613 and DKK1 to have great potential to serve as effective therapeutic targets for RA in clinical treatment, a lot more work must first be done. Our study contributed to the understanding of the RA pathogenesis mechanism, yet the full mechanism that involves various pathways needs further investigation.

## Abbreviations

FLSs: Fibroblast-like synoviocytes
miRNA: microRNA
MMP: Matrix metalloproteinase
qRT-PCR: quantitative real-time polymerase chain reaction.
RA: Rheumatoid arthritis
RASFs: Rheumatoid arthritis synovial fibroblasts
